# Synopsis of Address of Dean McEachran to the McGill Graduating Class

**Published:** 1895-05

**Authors:** 


### EDUCATIONAL.

### Synopsis of Address of Dean McEachran to the Graduating Class of McGill University

" When you decided on selecting this as your profession, no doubt your ideas of it were very different from what you have found it to be, in so far as the work imposed on you as students was concerned. No doubt you hesitated as to whether you would enter a school where you had to study for three years or one where you could graduate in two. Your experience has taught you that three years are all too short to accomplish your work. I must confess that as students you have my sympathy in your efforts to overtake so much work in so short a time, more especially when so much of that work is of a minute technical nature, for which you are not all equally adapted by nature or education. I refer to this for the double purpose of congratulating you on your having accomplished a degree of competency sufficient to enable you to graduate and to impress on your minds the necessity for your continuing to follow up the studies as graduates which you began as students. Our science, like all others, is a progressive one; we live in an age of progress. As electricity has revolutionized mechanical and applied science, so have the revelations of bacteriology revolutionized that of medicine, and the addition of a bacteriologist and a practical chemist to the staff of a well-appointed hospital is as necessary as is the compass to the navigator. No longer need the practitioner of any branch of medical science grope in the dark, going on, as during all these centuries he has, in blind faith. The laboratory, the microscope, the culture-room, and the experimental animal will determine accurately the nature of the infective microbe. From this new branch of medical science has evolved other progressive steps. The microbe causation of disease necessitated the discovery of medicinal agents with which to combat these by the prevention or destruction of them, hence the advance in surgery. Has not the necessity for antiseptic measures revolutionized practical surgery? Most undoubtedly. With a full knowledge of antiseptics the surgeon of to-day can, with confidence, perform operations which the surgeon of thirty years ago would have shrunk from undertaking. A knowledge of bacteriology has caused the physiologist and the histologist to waken up. We have long understood that blood consisted of liquor sanguinis, or plasma, and red and white corpuscles. It is but recently, however, that we have learned that there are four varieties of white corpuscles, two of which are known to be the enemies of the destructive bacilli, and it is to the presence of these, the mono- and polynucleus leucocytes that Capt. Schnikoff attributes the power of resisting disease. Not only have these branches of medical science been wakened up, but our whole domestic economy has been improved. You will thus see the absolute necessity for your continuing your studies. You ought to subscribe for and carefully read the professional journals and scientific magazines. Add to your libraries new professional books as they are issued up to date, and record and contribute to the journals the results of your observations and investigations. Those of you who can afford it ought to spend a year, at least, at one or more of the great European schools such as those of Germany, Russia, Austria, and France, where the teachers are specialists paid by the government to devote their time and energies to the work, not as is the case in British, American, and Canadian schools, where the teachers are men who require to attend to busy practices to enable them to support their families, and who tax their energies and overwork themselves in their efforts to do but scant justice to their professional duties. At such establishments, too, you will find equipments and appliances such as private self-supporting schools in English-speaking countries cannot afford, and the extent of the clinics in such large establishments will enable you to see in a few months a very large extent of clinical practice. Do not misunderstand me by inferring that I consider such schools better to study at from the beginning; they are not. I believe that the ground-work is better taught at the English-speaking schools; but for post graduate work I would recommend you to go to the European government establishments. It is to be hoped, however, that the day may yet come when this faculty will rival the other faculties of McGill in equipment, and then such advice will be unnecessary. When you go out to practice keep well in touch with the profession, and keep well in touch with your teachers. The more the faculty and the graduates are united and communicate with each other the stronger we will become, and the greater the influence your alma mater will have in bringing about those changes in matriculation, curriculum, and extension of time of study, which, I am happy to have learned, you yourselves consider to be necessary to enable undergraduates to satisfactorily accomplish the work which an advanced and advancing science demands. On behalf of your teachers and myself, and on behalf of the great university of which this faculty forms an integral part, I wish you Godspeed On their behalf I would beg of you to endeavor to realize the responsibility your graduation imposes upon you. Going forth in the world to carve your way, you do not do so as unknown, unrecognized, wandering, but you bear with you in your diplomas certificates of recognition and recommendation, guarantees to the whole world that you are honorable men, men worthy of trust, and men possessed of all the knowledge and attainments that you profess. If you would but endeavor to realize the responsibility your faculty and your examiners feel when they recommend you to receive the degree of this university you would see that we have a right to expect of you that by good conduct, unswerving fidelity to your God, your Queen and your alma mater, you will honor us by being an honor to yourselves and to your families. Bear in mind that you have a duty to perform to those poor dumb friends, to whose patient sufferings your professional duties will daily call you to minister. During your pupilage you have been taught to study comparative psychology, and have been led to think of your patients, not as men ordinarily think of them as senseless brute beasts, but as intelligent, sensitive, reasoning animals, but a little lower on the reasoning scale than ourselves, endowed with all the anatomical, physiological and psychological attributes which characterize our own bodies. Therefore avoid giving any unnecessary pain or discomfort to your patients during your efforts to cure their diseases, medically or surgically. Owing to the important changes that have been made in the curriculum of the medical college, more especially the extension of the session to nine months, this faculty is under the necessity of endeavoring to provide for special lectures and special demonstrations for its own students. Much inconvenience has been experienced in our efforts to take advantage of the medical classes for veterinary students, and especially has work of a practical nature in pharmacy and clinics been interfered with.

It is to be hoped that by some means or other the faculty will be enabled to carry out these improvements which have long been felt to be desirable, but circumstances now force them upon us as a necessity. I cannot close these remarks without referring with regret to a circumstance which, viewed in one light, may be a subject for congratulation. It must be a compliment to the university to have other great teaching bodies come to us for teachers of high standing, and if the New York Medical College succeeds in inducing Professor Mills to sever his connection with us, in the sense referred to, we must feel proud. I think, however, that in losing Prof. Mills we lose a scholarly man, a man of high moral standard, a man who has through his writings done much to spread the fame of McGill. His text-books on physiology and comparative physiology are in use in all the best English-speaking medical and veterinary colleges. Certainly this faculty will suffer the loss of a most competent, energetic and gifted colleague, and you, undergraduates a sincere friend and painstaking teacher. Let us hope that some steps will be taken to prevent McGill losing her Oslers and Mills. However, I am sure that whether he is taken from us or not the graduates and undergraduates of this faculty will ever hold his memory dear."

